# Experience with aortic arch inclusion technique using artificial blood vessel for type A aortic dissection: an application study

**DOI:** 10.1186/s13019-024-02741-8

**Published:** 2024-04-08

**Authors:** Qingfeng Li, Bin Li, Shuqiang Xi, Zhaobin Li, Zhe Zhu, Zeyue Jin, Fan Yang, Lei Liu

**Affiliations:** Department of Carvascular Surgery, Hebei Medical University Third Hospital, Shijiazhuang, Hebei Province China

**Keywords:** Type A aortic dissection, Aortic arch replacement, Stented elephant trunk, Surgery, Intra-aortic arch technique

## Abstract

**Background:**

This study aimed to elucidate the methodology and assess the efficacy of the aortic arch inclusion technique using an artificial blood vessel in managing acute type A aortic dissection (ATAAD).

**Methods:**

We conducted a retrospective review of 18 patients (11 males and 7 females, average age: 56.2 ± 8.6 years) diagnosed with ATAAD who underwent total aortic arch replacement (TAAR) using an artificial vascular “inclusion” between June 2020 and October 2022. During the operation, deep hypothermic circulatory arrest (DHCA) and selective antegrade cerebral perfusion (ACP) of the right axillary artery were employed for brain protection. The ‘inclusion’ total aortic arch replacement and stented elephant trunk (SET) surgery were performed.

**Results:**

Four patients underwent the Bentall procedure during the study, with one additional patient requiring coronary artery bypass grafting (CABG) due to significant involvement of the right coronary orifice. Three patients died during postoperative hospitalization. Other notable complications included two cases of postoperative renal failure necessitating continuous renal replacement therapy (CRRT), one case of postoperative double lower limb paraplegia, and one case of cerebral infarction resulting in unilateral impairment of the left upper limb. Eleven patients underwent computed tomography angiography (CTA) examinations of the aorta three months to one-year post-operation. The CTA results revealed thrombosis in the false lumen surrounding the aortic arch stent in seven patients and complete thrombosis of the false lumen around the descending aortic stent in eight patients. One patient had partial thrombosis of the false lumen around the descending aortic stent, and another patient’s false lumen in the thoracic and abdominal aorta completely resolved after one year of follow-up.

**Conclusions:**

Incorporating vascular graft in aortic arch replacement simplifies the procedure and yields promising short-term outcomes. It achieves the aim of total arch replacement using a four-branch prosthetic graft. However, extensive sampling and thorough, prolonged follow-up observations are essential to fully evaluate the long-term results.

## Background

Acute type A aortic dissection (ATAAD) is a severe disease characterized by sudden onset and rapid progression. After a definite diagnosis, surgical treatment should be performed as soon as possible [1]. ATAAD surgery is a high-risk operation with mortality rates as high as 12-20% [2–6]. In recent years, advances in medical technology, such as diagnostic tools and surgical procedures, have greatly facilitated the disease management of aortic dissection [7, 8]. The surgical treatment of aortic dissection is to resect the most severely damaged aortic segment and prevent blood from entering the false lumen, including the original tear of the aorta and subsequent tear segments [8]. However, the most suitable surgical method for treating ATAAD remains a point of significant domestic and international debate [9–11]. Total ascending aortic arch replacement with the frozen elephant technique is the classic method for treating type A aortic dissection [12, 13]. However, the traditional technique of four-branched prosthetic total aortic arch replacement (TAAR) presents difficulties in its implementation, primarily manifested in its complexity, multiple anastomoses required, long operation time, and high risk of bleeding.

In light of this, domestic and international scholars have been dedicated to finding improved methods for aortic arch reconstruction [14, 15]. We have adopted this aortic arch inclusion technique with a vascular graft to operate on ATAADs involving the arch and achieved good short-term clinical results. We will now summarize and report these results.

## Methods

### Patients

From June 2020 to October 2022, our hospital treated 18 patients with acute ATAAD using the aortic arch inclusion technique with artificial blood vessels. Among them were 11 males and 7 females, with ages ranging from 40 to 76 years old and an average age of 56.2 ± 8.6 years old. The patients’ associated conditions included long-term hypertension in 15 cases, diabetes in 3 cases, Marfan syndrome (MFS)in 2 cases, bicuspid aortic valve (BAV) malformation in 2 cases, moderate to severe aortic valve insufficiency in 5 cases, pericardial effusion in 2 cases, and unilateral lower limb artery ischemia in 2 cases; preoperative oliguria was present in 1 case. All cases were definitively diagnosed preoperatively via aortic CTA and bedside echocardiography. All patients underwent surgery within 24 h of arrival at the hospital.

### Surgical procedure

A midline thoracic incision was made to split the sternum and enter the chest, freeing the three brachiocephalic arteries. Right axillary artery, femoral artery double cannulation, and right atrial vein cannulation were performed to establish extracorporeal circulation. After clamping the ascending aorta, the ascending aorta was incised, and custodiol cardioplegic solution was directly perfused through the left and right coronary orifices to clear the thrombus in the dissection. Depending on the situation, a 5 − 0 Prolene double-headed needle with a spacer was used to suspend the junction of the valve leaflets; the aortic intima was transversely transected about 10 mm above the sinotubular junction. The aortic root is lined with a 10 mm wide polyester strip, and the reconstructed root is fixed and reinforced with 4 − 0 Prolene sutures using continuous mattress sutures. Pay attention to transmural sutures.

After the root treatment was finalized and the nasopharyngeal temperature reached 25℃, with the rectal temperature at 28℃, DHCA was initiated. The aortic clamp was then released, and selective antegrade cerebral perfusion (ACP) was carried out at 5–10 ml/kg/min via the right axillary artery cannula. The aortic arch was incised proximal to the orifice of the innominate artery, followed by probing of the aortic arch intima, confirming the presence of an intimal tear near the orifice of the brachiocephalic artery. Based on preoperative examination and intraoperative exploration, ‘inclusion’ total aortic arch replacement surgery was performed using a prosthetic blood vessel.

A Cronus intraoperative stent graft from Shanghai MicroPort Company of the appropriate size based on the true lumen diameter of the aortic arch was selected. The stent graft was positioned at the proximal level of the innominate artery ostium. Wedge resection of the opening of the brachiocephalic artery corresponds to part of the artificial blood vessel. Along the wedge incision in the aortic arch, continuous sutures with 5 − 0 Prolene thread were used to fix the stent graft to the surrounding aortic wall, ensuring that each stitch was transmural. The proximal orifice of the stent graft and the aortic wall near the orifice of the brachiocephalic artery (BCA) were continuously sutured intraluminally with 4 − 0 Prolene thread to fix and reinforce the arch incision, and a pad of about 5 mm wide was added to the wedge gap. This allowed the stent graft to cover all but about 1/4 of the cranial side of the proximal remnant intimal wall of the aortic arch.

After shaping and reinforcing the arch incision, a catheter with a water bladder was inserted into the stent’s artificial blood vessel, and 30 ml of saline was injected into the water balloon to restore lower body perfusion. Then, the shaped arch incision and the prosthetic blood vessel were continuously sutured with 4 − 0 Prolene thread to achieve end-to-end anastomosis. After the anastomosis was completed, lower body perfusion was temporarily stopped. The catheter was withdrawn, and then a clamp was used to clamp the prosthetic blood vessel to restore flow. Because, in some cases, the left subclavian artery (LSA) is far from the left common carotid artery (CCA), the left subclavian artery is not included in the wedge-shaped incision of the stent graft. After the distal anastomosis was completed, we sutured the proximal end of the left subclavian artery. Then, the distal end was anastomosed to the left common carotid artery with 5 − 0 Prolene thread to restore blood supply to the left subclavian artery.

**Fig. 1:**
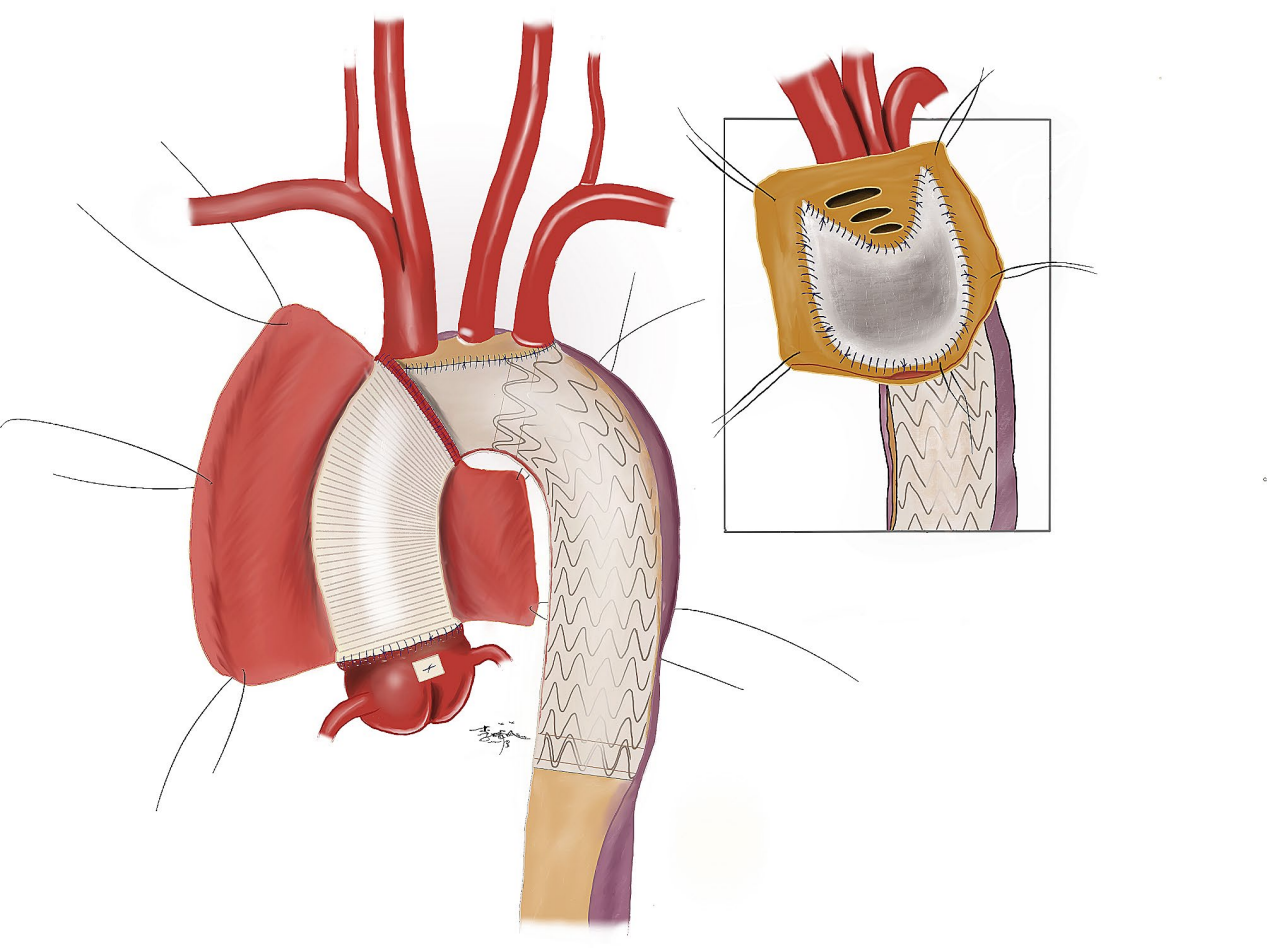
Aortic arch inclusion technique with artificial blood vessel

**Fig. 2:**
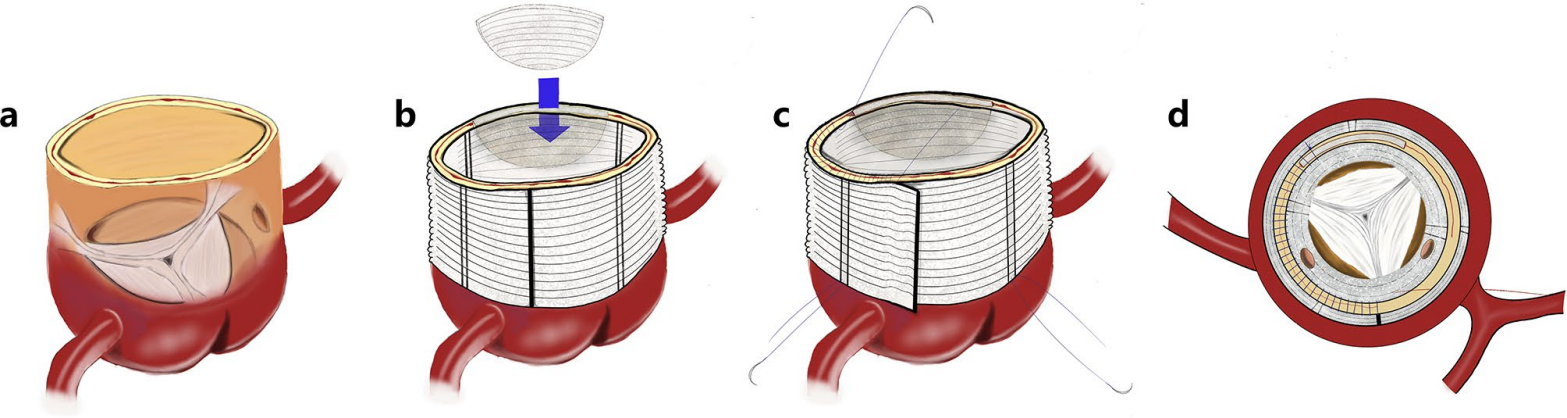
Schematic diagram of modified sandwich method of aortic root reinforcement. (**a**) Semi-perspective diagram of aortic root. (**b**) The aortic root is sutured and reinforced and a spacer is added. (**c**) Use the “modified sandwich” method for full-thickness transmural suturing (pay attention to fully exposing the left and right coronal openings). (**d**) Top view of aortic root after reinforcement

The proximal end of the prosthetic blood vessel was continuously sutured to the reshaped and reinforced aortic root with 3 − 0 Prolene thread for anastomosis. After de-airing, perfusion to the myocardium was resumed, and any bleeding was checked while circulation was ongoing. Bleeding points were reinforced with mattress sutures using a double-headed needle with a pad of 4 − 0 Prolene thread. Once there was no prominent bleeding, the residual aortic adventitia was sutured, and a shunt was established with the right atrium. (Figures 1 and 2)

### Follow-up

All discharged patients were followed up in the outpatient clinic or by telephone from 3 months to 1 year after the operation, and the occurrence of complications and prognosis were recorded.

### Statistical analysis

Continuous data are represented as means ± standard deviations, and categorical data are represented as frequencies (percentages). The data were analyzed using SPSS software V20.0 for Windows (IBM Corporation, New York, USA).

## Results

### Operative data

In our group, 18 patients with acute ATAAD underwent artificial blood vessel “endovascular” total arch replacement surgery, of which 4 underwent Bentall surgery concurrently, and 1 underwent coronary artery bypass grafting due to severe involvement of the right coronary artery (RCA) opening. In operation, the intimal tear was located in the ascending aorta in 11 cases and on the minor curve side of the aortic arch and proximal descending aorta in 7 cases. In 5 patients, the openings of the three brachiocephalic vessels were far away, or the opening of the left subclavian artery was difficult to expose (Table 1). The length of the avascular area of the stent was insufficient during the operation. Including all three brachiocephalic blood vessel openings in the island anastomosis is impossible. Therefore, an end-to-side anastomosis was performed between the left subclavian and the left common carotid arteries.

**Table 1:** Preoperative clinical characteristics

Characteristic	n(%)
Age	56.2 ± 8.6
Patients	18
Male/female	11/7(61.1/38.9)
Comorbidities	
Hypertension	15(83.3)
Diabetes mellitus	3(16.7)
Coronary heart disease	1(5.6)
Cerebrovascular disease	1(5.6)
Marfan syndrome	2(11.1)
Primary entry	
Ascending aorta	13(72.2)
Arch	2(11.1)
Multiple entry	1(5.6)
No entry found	1(5.6)
Malperfusion	
Coronary	5(27.8)
Leg	7(38.9)
Cerebral	1(5.6)

### Mortality and morbidity

During the postoperative hospital stay, three deaths occurred. One patient died of sudden cardiac arrest due to postoperative coronary artery complications caused by residual root lesions. Another patient died from a massive hemorrhage due to the tearing of the adventitia of the residual false cavity in the root. The third patient died of multiple organ failure due to renal failure and infection following CRRT. Other significant complications included two instances of postoperative renal failure that required CRRT, one instance of postoperative paraplegia in both lower limbs (with sensory level reaching both knees at discharge and no significant improvement during the 1-year follow-up) and one case of postoperative cerebral infarction resulting in unilateral physical impairment of the left upper limb (which mostly recovered after six months of follow-up). Mortality and postoperative morbidity are summarized in Table 2.

**Table 2:** Surgical procedure, interaoperative and postperative data

Variables	Data
Operative time (min)	540.0 ± 114.0
CPB time (min)	251.3 ± 90.6
Clamp time (min)	147.5 ± 64.9
Antegrade cerebral perfusion time (min)	21.0 ± 13.6
Hospital mortality	3(16.7%)
Postoperative complications	
Paraplegia	1(5.6%)
Stroke	1(5.6%)
AKI requiring dialysis	3(16.7%)
Postoperative ICU stay(d)	8.56 ± 9.2
24 h Postoperative Drainage (ml)	537.5 ± 322.1
Intubation time (d)	5.7 ± 5.5
Reintubation	2(11.1%)
Tracheotomy	2(11.1%)
Concomitant procedures	
Bentall procedure	4(22.2%)
Coronary artery repair	1(5.6%)

### Follow-up data

**Fig. 3:**
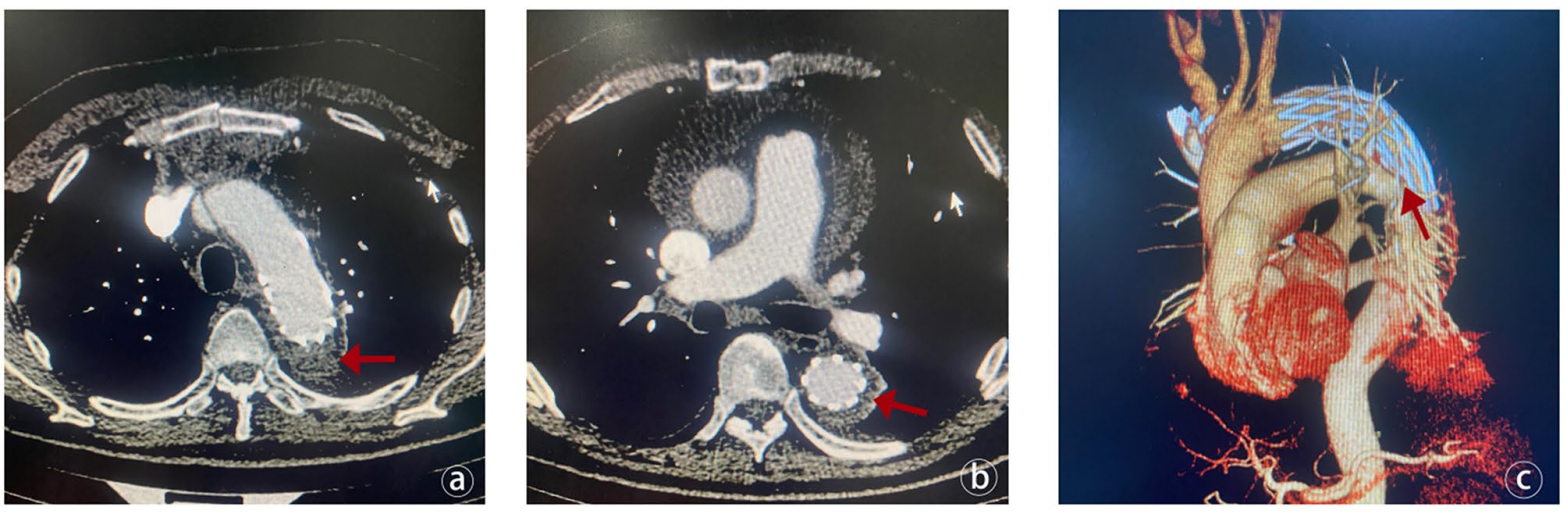
Assessment of stent and thrombus status after TAAR. (**a**) The red arrow indicates that the thrombus in the false lumen of the aortic arch has been thrombosed after surgery, and the stent is in good shape. (**b**) The yellow arrow indicates that the thrombus in the false lumen of the descending aortic stent has been completely thrombosed after surgery, the stent is in good shape, and there is no endoleak. (**c**) The 3D reconstruction of the patient’s aortic CTA demonstrates the morphology of the stent

All discharged patients had outpatient or telephone follow-up from 3 months to 1 year after operation. During the follow-up period, there was no death or reoperation. Eleven patients underwent aortic CTA. Among them, one patient had complete absorption of the entire thoracic and abdominal aortic dissection, seven had complete thrombus formation within the false lumen around the aortic arch stent, 1 had partial thrombus formation in the false lumen around the descending aortic stent, and 8 had complete thrombosis in the false lumen around the descending aortic stent (Fig. 3).

## Discussion

For treating ATAAD, most scholars prefer to excise the ascending aorta or the primary tear in the arch for most cases and perform distal open anastomosis under deep hypothermic circulatory arrest. The rationale for this approach is to resolve the main issue with the most straightforward surgical method, help patients through the most critical period, and prioritize patient survival. However, for ATAADs, primarily when the tear is located at the far end of the arch, there are still risks associated with merely replacing the ascending aorta or a combination of the ascending aorta and hemiarch. The risks of tear expansion and bleeding from the distal anastomosis caused by false lumen reperfusion should not be overlooked [15–18]. By placing a stent graft inside the descending aorta, the intimal tear inside the vessel can be closed, and the true lumen, which is under pressure, can be maximally expanded. In addition, the false lumen can be compressed and eliminated, allowing the torn vascular wall structure to reattach and reconstruct the vascular wall [19, 20]. This technique reduces the risk of patients needing reoperation in the future and, by eliminating the residual false lumen near the arch anastomosis, reduces false lumen reperfusion, strengthens the solidity of the distal anastomosis, and thus lowers the risk of surgical bleeding. ATAAD surgery has been widely implemented using this technique, becoming the preferred surgical method for most cases of acute ATAAD in our country [20]. However, using a four-branch artificial vessel for total arch replacement has some disadvantages. Because it requires dissection and release of the arch and head-arm vessels, the process of anastomosis and hemostasis for the three head-arm vessels is time-consuming and laborious. These factors increase the time for extracorporeal circulation and operation [21]. In order to simplify total arch replacement surgery, Chinese scholars have conducted extensive practice and exploration, including the arch debranching Hybrid technique [22], intraoperative stent technique with branches [23], distal sutureless technique, and the “intima-included” aortic arch replacement technique with vascular graft [22]. The artificial vessel “intima-included” aortic arch replacement technique designed by Professor Ke Xiang Liu’s team at Jilin University simplifies the traditional four-branch artificial vessel total arch replacement to the proximal arch anastomosis. It avoids the meticulous and complicated dissection of three-head-arm vessels. Moreover, the traditional four-branch vessel total arch replacement combined with frozen elephant trunk surgery was realized to eliminate the arch and descending aorta proximal breach, reinforce the distal anastomosis, reduce the distal reoperation rate, and reduce the risk of anastomotic bleeding.

Compared with using a four-branch artificial vessel for arch replacement surgery, this method reduces the range of arch and head-arm artery dissection and the number of anastomoses, simplifying the surgical operation and reducing the risk of bleeding. Using this technique, the plane of arch anastomosis is moved forward to the proximal end of the innominate artery opening, making it easier to expose and easier to anastomose. To facilitate hemostasis, all cases in this group used the residual tumor wall for wrapping and right atrium shunting. Since this technique does not use a four-branch artificial vessel, wrapping and shunting are easier to implement, helping to reduce postoperative drainage. At the same time, this technique also saves the cost of a four-branch artificial vessel, reducing the economic burden on patients. The island anastomosis between the opening of the brachiocephalic artery and the artificial blood vessel is carried out in the cavity of the aortic arch. Compared with the traditional island anastomosis technique, the risk of bleeding on the posterior wall of the island anastomosis is avoided [22, 23]. The operation only increases the arch island anastomosis time by a few minutes under deep hypothermic circulatory arrest during distal open anastomosis, compared with the excision of the ascending aorta or the primary tear in the arch. This technique is more convenient and safer than hemiarch replacement surgery. While incorporating the advantages of total arch replacement and elephant trunk surgery, it achieves the principle of simplifying surgery as much as possible, as proposed by international colleagues.

Notably, Eugenio Neri raised the “intimal re-layering technique” to simplify arch reconstruction. Although the paper showed complete thrombosis in the dissected arch in all patients, the intimal re-layering technique may not be feasible in overt arch aneurysms because it is not truly arch exclusion from circulation [24, 25]. Recently, the intraoperative stent graft utilized by Professor Ke Xiang Liu’s team is a specially designed intraoperative stent graft with a non-vascular area length exceeding 5 cm at the proximal end. We employed Cronus intraoperative stent grafts from MicroPort, which feature a stent-free area length of 2 cm at the proximal end. In five cases, due to the considerable distance between the opening of the left subclavian artery and the opening of the left common carotid artery, the difficulty in exposing it, including the left subclavian artery in the island, was challenging. Therefore, we relocated the opening of the left subclavian artery to the left common carotid artery after completing the arch anastomosis and restoring circulation.

The “intima-included” aortic arch replacement surgery has certain limitations, with the most concerning issues being leaks at the distal anastomosis and closure of the arch false lumen. Professor Ke Xiang Liu’s report of 154 long-term cases [14] showed that at discharge, the closure rate of the aortic arch false lumen was 77.8%, and the thrombus formation rate in the descending aorta was 69.2%. Six months after surgery, the aortic arch false lumen closure rate reached 92.4%, and the descending aorta thrombus formation rate was 74.3%. To reduce the risk of intraoperative leakage, when the aortic arch wall is anastomosed to the stent graft in an island fashion, it is necessary to ensure that the suture needle thoroughly penetrates the aortic adventitia and take care to prevent intimal tears caused by needle holes.

### Limitations

This single-center retrospective study was characterized by a short follow-up period and a limited sample size. Computed tomography angiography (CTA) data from the pre-discharge and one-year follow-up phases were unavailable for all patients. Detailed long-term results and a comprehensive analysis will be reported in a subsequent publication.

## Conclusion

Our modified aortic arch incorporation technique for treating acute type A aortic dissection (ATAAD) is safe and feasible. This technique simplifies the surgical procedure, facilitating its widespread adoption and enabling surgeons to manage ATAAD more efficiently. While the short-term efficacy is encouraging, further study is needed to evaluate long-term efficacy, including its impact on descending aortic remodeling and the prevention of pseudoaneurysm formation.

## Data Availability

Data are available from the authors upon request.

## Abbreviations

ACP: antegrade cerebral perfusion
ATAAD: acute type A aortic dissection
BAV: bicuspid aortic valve
BCA: brachiocephalic artery
CABG: coronary artery bypass grafting
CCA: left common carotid artery
CRRT: continuous renal replacement therapy
CTA: computed tomography angiography
DHCA: deep hypothermic circulatory arrest
LSA: left subclavian artery
MFS: Marfan syndrome
SCP: selective cerebral perfusion
SET: stented elephant trunk
TAAR: total aortic arch replacement
RCA: right coronary artery
