# Correction: Experience with aortic arch inclusion technique using artificial blood vessel for type A aortic dissection: an application study

**DOI:** 10.1186/s13019-024-02774-z

**Published:** 2024-04-20

**Authors:** Qingfeng Li, Bin Li, Shuqiang Xi, Zhaobin Li, Zhe Zhu, Zeyue Jin, Fan Yang, Lei Liu

**Affiliations:** Department of Carvascular Surgery, Hebei Medical University Third Hospital, Shijiazhuang, Hebei Province China

**Correction: J Cardiothorac Surg 19, 189 (2024)**

**https://doi.org/10.1186/s13019-024-02741-8**

Following the publication of the original article [1], Figs 2 and 3 were wrongly published; The figure published as Fig. 3 should actually be Fig. 2. Additionally, the figure originally intended to be presented as Figure 3, which contains vital imaging data pertinent to the study was missing. The figures 2 and 3 should have appeared as shown below.

**Fig. 2:**
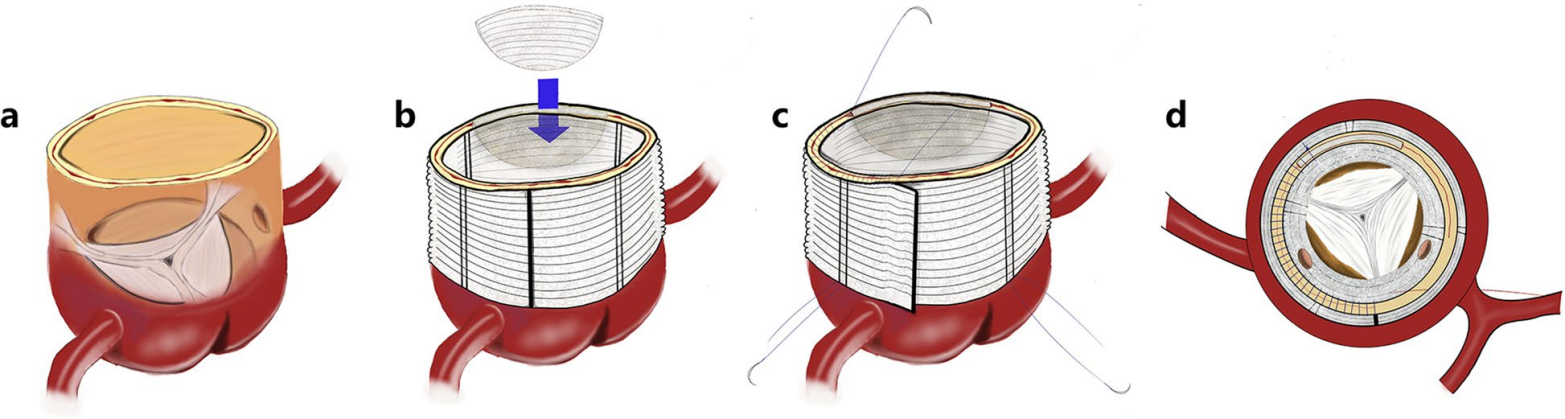
Schematic diagram of modified sandwich method of aortic root reinforcement. (**a**) Semi-perspective diagram of aortic root. (**b**) The aortic root is sutured and reinforced and a spacer is added. (**c**) Use the “modified sandwich” method for full-thickness transmural suturing (pay attention to fully exposing the left and right coronal openings). (**d**) Top view of aortic root after reinforcement

**Fig. 3:**
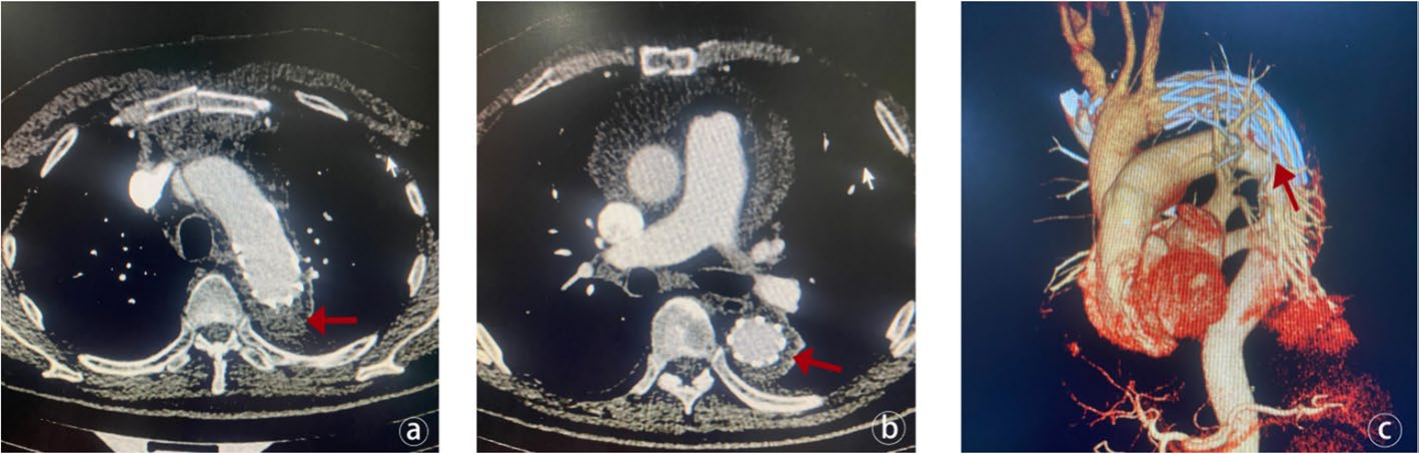
Assessment of stent and thrombus status after TAAR. (**a**) The red arrow indicates that the thrombus in the false lumen of the aortic arch has been thrombosed after surgery, and the stent is in good shape. (**b**) The yellow arrow indicates that the thrombus in the false lumen of the descending aortic stent has been completely thrombosed after surgery, the stent is in good shape, and there is no endoleak. (**c**) The 3D reconstruction of the patient’s aortic CTA demonstrates the morphology of the stent

The original article has been corrected.

## References

[1] Li Q, Li B, Xi S (2024). Experience with aortic arch inclusion technique using artificial blood vessel for type A aortic dissection: an application study. J Cardiothorac Surg.

